# Effects of *Bellamya* Stocking Density on Water Quality and Bacterial Community Responses in Aquaculture Effluent

**DOI:** 10.3390/microorganisms14081813

**Published:** 2026-08-17

**Authors:** Hao Zhu, Huichao Shen, Fan Wu, Xuan Che, Jiahua Zhang

**Affiliations:** 1 Fishery Machinery and Instrument Research Institute, Chinese Academy of Fishery Sciences, No. 63 Chifeng Road, Yangpu District, Shanghai 200092, China; zhuhao@fmiri.ac.cn (H.Z.); chexuan@fmiri.ac.cn (X.C.); zhangjiahua@fmiri.ac.cn (J.Z.); 2 East China Sea Fisheries Research Institute, Chinese Academy of Fishery Sciences, Shanghai 200090, China; 3 Key Laboratory of Aquatic Nutrition and Feed Science of Jiangsu Province, College of Animal Science and Technology, Nanjing Agricultural University, No. 1 Weigang Road, Nanjing 210095, China; shc2396554353@163.com; 4 Key Laboratory of Aquaculture Facilities Engineering, Ministry of Agriculture and Rural Affairs, Shanghai 200092, China

**Keywords:** *Bellamya*, stocking density, aquaculture effluent, water quality, bacterial community

## Abstract

Aquaculture effluent commonly contains suspended solids, inorganic nitrogen, reactive phosphate and algal biomass, creating a need for low-input ecological treatment approaches. This study assessed endpoint water-quality and bacterial-community responses to *Bellamya* stocking density in aquaculture effluent. A no-snail control (CON) and four stocking-density treatments (LD, MD, MHD and HD) were established with three independent tank replicates per treatment. Suspended solids (SS), NH_4_^+^-N, NO_2_^−^-N, NO_3_^−^-N, PO_4_^3−^-P and chlorophyll a (Chl-a) were quantified, and bacterial communities were characterized by 16S rRNA gene sequencing of the V3–V4 region. At the 60-day endpoint, all *Bellamya*-stocked treatments had lower NH_4_^+^-N and NO_3_^−^-N concentrations than the control, and MHD and HD also had lower PO_4_^3−^-P and Chl-a. SS showed a nonsignificant downward tendency, whereas NO_2_^−^-N showed a nonsignificant upward tendency. Bray–Curtis NMDS visualized treatment-associated separation, and PERMANOVA detected significant differences among treatments (R^2^ = 0.62, *p* = 0.001; stress = 0.0822). However, PERMDISP was also significant (F_4,10_ = 4.99, *p* = 0.001), indicating heterogeneous within-treatment dispersion and requiring cautious interpretation of the PERMANOVA result. Representative genera showed distinct treatment-associated abundance patterns, and 46 of 90 genus–environment associations remained significant after Benjamini–Hochberg correction. Predicted KEGG Level 3 pathways varied numerically among treatments, but none remained significant after false-discovery-rate correction. These findings indicate that *Bellamya* stocking may provide a low-input ecological component of aquaculture-effluent management, while the microbiome results should be interpreted as community-level associations and predicted functional trends rather than evidence of microbial causality or pathway activation.

## 1. Introduction

Aquaculture intensification increases the discharge of water containing suspended particles, inorganic nutrients and algal biomass, thereby increasing eutrophication risk [1,2,3]. Because aquaculture effluents are usually produced in large volumes at relatively low pollutant concentrations, treatment must balance removal efficiency with energy use, land demand and operational cost. Biological and nature-based approaches are therefore being developed as lower-input complements to conventional physicochemical treatment [2,3,4,5].

Microorganisms are central to the fate of nutrients in aquaculture waters. Ammonia oxidation, nitrite oxidation, denitrification and anammox collectively determine the transformation and removal of inorganic nitrogen, while microbial processing of dissolved and particulate organic matter can influence phosphorus availability and algal–bacterial interactions [6,7,8,9,10]. These processes are environmentally sensitive and may become uncoupled under changing oxygen, carbon and nutrient conditions. In particular, NO_2_^−^-N can accumulate when ammonia oxidation proceeds faster than nitrite oxidation or subsequent reduction processes [6,7]. Accordingly, the assessment of ecological effluent treatment should combine conventional water-quality indicators with an analysis of bacterial community structure and taxon–environment relationships.

Compared with treatment systems that depend on continuous aeration, chemical dosing or extensive infrastructure, benthic-gastropod-mediated treatment may require relatively little external energy input and can simultaneously modify suspended particles, algal biomass, sediment–water exchange and microbial habitat through grazing, particle ingestion, biodeposition and bioturbation [11,12]. *Bellamya* was selected as a locally available freshwater benthic grazer with the potential to influence several of these processes. Because most previous work has focused on sediments or host-associated compartments, direct evaluation of water-column responses in aquaculture effluent remains limited.

Previous studies indicate that the ecological effects of *Bellamya* depend on species identity and habitat compartment. In *Bellamya* purificata, stocking density and bioturbation altered sediment organic-matter degradation, nitrogen-form distribution and the assembly of bacterial and other microbial communities [13,14,15,16,17]. Studies of *Bellamya* aeruginosa further showed that cyanobacterial-bloom exposure and association with submerged macrophytes were accompanied by shifts in gut and epiphytic bacterial communities [18,19]. Because species-level morphological or molecular confirmation was unavailable for the animals used in the present experiment, they are conservatively designated *Bellamya* sp. throughout the manuscript.

Evidence from other integrated aquaculture systems also supports coupling physicochemical assessment with microbial-community analysis. Algae–bacteria biofilms used for tailwater treatment under sulfamethoxazole stress have been shown to affect nutrient-removal performance, microbial-community structure and antibiotic-resistance-gene profiles [20]. Similarly, integrated rice–fish culture can reshape bacterioplankton composition and community assembly [21]. Although these systems differ mechanistically from *Bellamya* stocking, they illustrate the value of evaluating ecological interventions using coordinated water-quality and microbiological endpoints.

High-throughput 16S rRNA gene sequencing can characterize bacterial alpha diversity, beta diversity, taxonomic composition and treatment-associated taxa, while ordination, LEfSe and taxon–environment analyses provide complementary community-level evidence [22,23,24,25,26,27,28,29]. Because taxonomic identity does not uniquely determine ecological function, 16S-based functional prediction was treated as hypothesis-generating inference rather than the direct measurement of functional genes, transcriptional activity or pathway flux [30,31,32].

The present study therefore investigated the effects of *Bellamya* stocking density on aquaculture effluent using an integrated water-quality and bacterial-community framework. We hypothesized that *Bellamya* stocking density would alter nutrient and algal-biomass indicators and that these changes would be accompanied by shifts in bacterial community structure. Specifically, we aimed to (i) evaluate stocking-density effects on SS, inorganic nitrogen, reactive phosphate and Chl-a; (ii) characterize bacterial alpha and beta diversity and taxonomic composition; (iii) identify treatment-responsive taxa and representative genera; and (iv) assess associations between selected bacterial genera and water-quality variables using Spearman correlation and redundancy analysis (RDA).

## 2. Materials and Methods

### 2.1. Experimental Design and Sample Collection

The experiment comprised five treatments: a no-snail control (CON) and four *Bellamya* stocking-density treatments, designated LD, MD, MHD and HD. Each treatment included three independent tanks (*n* = 3). The CON treatment contained no *Bellamya*, whereas 15, 25, 30 and 65 individuals were stocked in the LD, MD, MHD and HD treatments, respectively. Because these levels were not evenly spaced, stocking density was treated as a categorical experimental factor rather than as a continuous dose variable. Based on the 10.2 L water volume, numerical stocking densities were 1.47, 2.45, 2.94 and 6.37 ind. L^−1^ for LD, MD, MHD and HD, respectively. All tanks received aquaculture effluent from the same source and were maintained under the same experimental conditions apart from *Bellamya* stocking density.

The experiment was conducted in transparent acrylic tanks measuring 30 cm × 17 cm × 20 cm. Each tank contained 10.2 L (0.0102 m^3^) of aquaculture effluent collected from a culture pond in Songjiang, Shanghai, China. Stocking number, initial total wet weight, water volume, numerical stocking density and biomass stocking density are summarized in Table 1. The snails were collected from a crab pond, identified only to genus level and designated *Bellamya* sp. Individuals were visually selected to minimize conspicuous size variation; however, shell length and individual wet-weight distributions were not recorded. For descriptive comparison, numerical stocking density was calculated as the number of snails per tank divided by 10.2 L, and calculated mean wet mass per stocked individual was obtained by dividing the treatment mean initial wet weight per tank by stocking number. The resulting estimates ranged from 3.81 to 4.41 g ind.^−1^ and represent treatment-level stocking mass rather than directly measured individual size distributions. Before the experiment, shell surfaces were rinsed, and the snails were held for 24 h in tap water followed by 24 h in filtered pond water to reduce residual gut contents and acclimate them to the experimental water. The experiment lasted 60 days, from 1 September to 30 October 2025, and water temperature ranged from 15 to 25 °C.

At the end of the experiment, water was collected independently from each experimental unit for physicochemical analysis and 16S rRNA gene sequencing. For microbial DNA sampling, 5 L of water from each tank were sequentially filtered through 5.0 μm and 0.22 μm membranes. The membrane samples were collected aseptically and used for subsequent DNA extraction.

### 2.2. Water-Quality Measurements

Suspended solids (SS) were determined gravimetrically according to GB 11901-1989 [33]. Briefly, 50 mL of thoroughly mixed water was filtered through a 0.45 μm membrane, and the retained material was dried at 103–105 °C to a constant weight.

Ammonium nitrogen (NH_4_^+^-N) and nitrate nitrogen (NO_3_^−^-N) were determined according to HJ 535-2009 [34] and HJ/T 346-2007 [35], respectively, using a DT-3900 multiparameter water-quality analyzer (Beijing Huamei Water Analytical Instrument Technology Co., Ltd., Beijing, China).

Nitrite nitrogen (NO_2_^−^-N) was measured spectrophotometrically according to GB 7493-1987 [36]. Nitrite was diazotized with sulfanilamide and coupled with N-(1-naphthyl)-ethylenediamine dihydrochloride, and absorbance was measured at 540 nm.

Reactive phosphate (PO_4_^3−^-P) was determined using the molybdenum blue method according to GB 17378.4-2007 [37]. Water samples were filtered through a 0.45 μm membrane and reacted with ammonium molybdate and ascorbic acid, and absorbance was measured at 882 nm.

Chlorophyll a (Chl-a) was determined spectrophotometrically according to HJ 897-2017 [38]. Algal biomass was retained on glass-fiber filters and extracted with 90% acetone, and absorbance was measured at 750, 664, 647 and 630 nm. Measurements from each tank represented one biological replicate.

### 2.3. DNA Extraction, PCR Amplification and Sequencing

Total microbial DNA was extracted from the membrane samples using a HiPure Water DNA Kit (Magen Biotechnology Co., Ltd., Guangzhou, China; Cat. No. D3145-02) according to the manufacturer’s instructions. DNA concentration and purity were assessed using a NanoDrop 2000 spectrophotometer (Shanghai, China), and DNA integrity was evaluated by 1% agarose gel electrophoresis at 5 V cm^−1^ for 20 min.

The V3–V4 region of the bacterial 16S rRNA gene was amplified using barcode-tagged primers 338F (5′-ACTCCTACGGGAGGCAGCAG-3′) and 806R (5′-GGACTACHVGGGTWTCTAAT-3′). PCR amplification was performed in a 20 μL reaction mixture containing 4 μL of 5× FastPfu Buffer, 2 μL of 2.5 mM dNTPs, 0.8 μL of each primer (5 μM), 0.4 μL of FastPfu Polymerase, 0.2 μL of bovine serum albumin (BSA), approximately 10 ng of template DNA, and nuclease-free water to a final volume of 20 μL.

The thermal cycling program consisted of an initial denaturation at 95 °C for 3 min; 30 cycles of denaturation at 95 °C for 30 s, annealing at 55 °C for 30 s, and extension at 72 °C for 45 s; followed by a final extension at 72 °C for 10 min and holding at 10 °C.

Three independent PCR reactions were performed for each sample, and the resulting amplicons were pooled. PCR products were evaluated by 2% agarose gel electrophoresis and purified using an AxyPrep DNA Gel Extraction Kit (Axygen Biosciences, Union City, CA, USA). Together with NanoDrop assessment and DNA-integrity examination, these procedures formed part of the sequencing provider’s routine DNA and PCR quality-control workflow. An extraction blank was not included in the original DNA-extraction workflow. According to the sequencing provider, a batch-level no-template PCR control, designated CK in the provider’s routine quality-control report, was included during PCR amplification and examined by agarose gel electrophoresis. No visible amplicon band was observed in the CK lane. The CK control was not carried forward to library preparation or sequencing. Purified amplicons were quantified using a Quantus™ Fluorometer (Promega, Madison, WI, USA) and pooled in proportions appropriate for the intended sequencing depth.

Sequencing libraries were prepared using the Hieff NGS^®^ Ultima DNA Library Prep Kit for Illumina^®^ (Yeasen Biotechnology, Shanghai, China) according to the manufacturer’s instructions. Paired-end sequencing (2 × 250 bp) was performed on an Illumina MiSeq platform.

### 2.4. Sequence Processing and Taxonomic Annotation

Raw paired-end reads were demultiplexed according to sample-specific barcodes. Quality filtering was performed using the sequencing-provider pipeline with Trimmomatic [39], FLASH and USEARCH. Reads were truncated when the mean quality score within a 10 bp sliding window fell below 20, and reads shorter than 50 bp after trimming were discarded. Paired-end reads were merged using FLASH [40] with a minimum overlap of 10 bp; merged sequences with an overlap mismatch ratio greater than 0.2 were removed. Barcode mismatches were not allowed, and no more than two primer mismatches were permitted. Chimeric sequences were removed using USEARCH in combination with de novo and reference-based detection against the GOLD reference database [41].

High-quality sequences were dereplicated, singleton sequences were removed, and the remaining unique sequences were clustered into operational taxonomic units (OTUs) at 97% sequence similarity using USEARCH v7.0.1090 and the UPARSE workflow [23]. All quality-filtered sequences were subsequently mapped back to representative OTU sequences at ≥97% similarity to generate the OTU abundance table. Taxonomic assignment of representative sequences was performed using RDP Classifier v2.2 implemented in QIIME v1.9.0 against the SILVA Release 132 database [22,24,25], with a confidence threshold of 0.7. All downstream taxonomic analyses were therefore based on 97%-similarity OTUs rather than amplicon sequence variants.

The sequencing-provider workflow retained both the original and normalized OTU tables. The normalized OTU table was obtained by rarefying all samples to the minimum sequencing depth across the dataset and was used for taxonomic composition, beta-diversity, and differential-taxon and taxon–environment analyses. Alpha-diversity indices and rarefaction curves were obtained from the provider-generated 97%-similarity OTU output.

### 2.5. Alpha Diversity, Community Structure and Taxonomic Composition

Alpha diversity was evaluated at 97% OTU similarity using mothur v1.41.0 and included ACE and Chao1 richness estimators, Shannon and Simpson diversity indices, and Good’s coverage [26,42,43,44]. Rarefaction curves based on observed OTU richness were generated to assess sequencing sufficiency. Chao1 and Shannon indices were selected for visualization in the main text, whereas the complete alpha-diversity results are provided in Supplementary Table S1.

Between-sample community differences were quantified using Bray–Curtis dissimilarity calculated from the normalized OTU table [45]. Non-metric multidimensional scaling (NMDS) and PERMANOVA (adonis2) were performed in the R package vegan with 999 permutations [27]. Homogeneity of multivariate dispersion was assessed using PERMDISP on the same Bray–Curtis distance matrix. Distances from individual samples to their treatment-group spatial medians were calculated with betadisper (type = “median”), and significance was evaluated with 999 permutations. Sample distances are visualized in Supplementary Figure S4.

Bacterial community composition was summarized from the normalized OTU table at the phylum and genus levels. Dominant phyla were displayed in the main text. The top 20 genus profile was retained as a supplementary overview because numerous low-abundance genera were grouped within the “Others” category. Five named genera identified among the LEfSe-discriminative taxa—*Rhodobacter*, *Ahniella*, *Novosphingobium*, *Sphingobacterium* and *Silanimonas*—were selected for illustrative sample-level visualization. Selection was based on unambiguous genus-level annotation, sufficient abundance for visualization, and distinct, nonredundant treatment-associated patterns. The subset was intended as a descriptive illustration rather than a statistical ranking or an exhaustive set of responsive taxa. Ecological relevance was evaluated only after selection by comparing abundance patterns with the measured water-quality gradients. All 25 LEfSe-identified discriminative taxa, including *Legionella* and *Rubrivivax*, are reported in Supplementary Table S2.

### 2.6. Differential Taxa, Taxon–Environment Associations and Functional Prediction

Differentially enriched bacterial taxa among treatments were identified using linear discriminant analysis effect size (LEfSe) [28]. The final five-group analysis was performed using the Galaxy implementation of LEfSe. The Kruskal–Wallis test was used to screen taxa showing significant differences among groups, followed by linear discriminant analysis to estimate the effect size of each discriminative feature. Taxa with *p* < 0.05 and an LDA score ≥ 3.0 were retained as discriminative features. The term “taxa” is used because the significant LEfSe output included named genera together with uncultured, unclassified, no-rank and higher-level lineages.

For focused exploratory taxon–environment analysis, a fixed panel of 15 named genera was defined before the Spearman and RDA analyses. Each genus was detected in all 15 samples, had an overall mean relative abundance of at least 0.1%, and showed a treatment-responsive abundance pattern in the fixed 15-genus Kruskal–Wallis screen after Benjamini–Hochberg correction; LEfSe support was documented but was not required for inclusion. The complete quantitative evidence, including overall abundance, prevalence, treatment means, univariate statistics and LEfSe support, is provided in Supplementary Table S4. The panel comprised *Rhodobacter*, *Sphingomonas*, *Novosphingobium*, *Bradyrhizobium*, *Ahniella*, *Flavobacterium*, *Acinetobacter*, *Diaphorobacter*, *Comamonas*, *Sphingobacterium*, *Pelomonas*, *Tabrizicola*, *Silanimonas*, *Labrys* and *Hirschia* and was treated as a predefined exploratory panel rather than a set of proven functional or causal bacteria. Spearman correlations were calculated across all 15 sample-level observations between genus relative abundances and SS, NH_4_^+^-N, NO_2_^−^-N, NO_3_^−^-N, PO_4_^3−^-P and Chl-a. All 90 *p*-values were adjusted jointly using the Benjamini–Hochberg procedure, and heatmap significance symbols were based on the resulting q-values.

Redundancy analysis (RDA) was performed in R using the vegan package. The sample-level relative-abundance matrix of the 15 selected genera was Hellinger-transformed, and the six water-quality variables were z-score-standardized. Overall and axis-wise significance was evaluated using 999 unrestricted permutations with a fixed random seed. Marginal effects of the six environmental variables were evaluated by permutation tests; the six resulting *p*-values were adjusted as one Benjamini–Hochberg family, and variance inflation factors (VIFs) were calculated as collinearity diagnostics.

Potential bacterial functional profiles were inferred using PICRUSt2 [29]. According to the sequencing provider, functional prediction was based on a reference genome set derived from 41,926 bacterial and archaeal genomes in the Integrated Microbial Genomes (IMG) database, using the snapshot dated 8 November 2017. KEGG Orthology (KO) and COG functional assignments were based on annotations integrated with this IMG-derived reference genome set; therefore, no separate standalone KEGG or COG database release was specified by the sequencing provider. The provider-generated KEGG Level 2 and Level 3 abundance tables were subsequently used for downstream analysis. These predictions were treated as functional inferences rather than direct measurements of functional-gene abundance, transcription, enzyme activity or pathway flux [30,31,32]. Predicted KEGG Level 2 and Level 3 abundances were converted to within-sample relative abundances before visualization and group comparison. Among 328 Level 3 pathways, 22 all-zero or invariant pathways were excluded, and the remaining 306 pathways were compared among treatments using Kruskal–Wallis tests. Raw *p*-values from all 306 tests were adjusted jointly using the Benjamini–Hochberg procedure. A concise statistical summary is provided in Supplementary Table S6, whereas complete pathway-level treatment means, Kruskal–Wallis H statistics, raw *p*-values and Benjamini–Hochberg-adjusted q-values are provided in Supplementary Data S1.

### 2.7. Statistical Analysis

Univariate analyses were performed using IBM SPSS Statistics version 27.0, and the figures were prepared using GraphPad Prism version 9.5.0. Data are presented as mean ± SEM, with individual tank-level biological replicates displayed where applicable. Normality and homogeneity of variance were assessed using the Shapiro–Wilk and Levene tests, respectively. One-way ANOVA followed by Tukey’s test was used when parametric assumptions were satisfied; otherwise, the Kruskal–Wallis test followed by Dunn’s multiple-comparison procedure was applied. Alpha-diversity indices were compared using Kruskal–Wallis tests. PERMANOVA and PERMDISP were based on the same normalized OTU table and Bray–Curtis distance matrix and used 999 permutations. For the 90 Spearman tests, significance symbols in the heatmap and Supplementary Table S3 were based on the same Benjamini–Hochberg-adjusted thresholds (* q < 0.05, ** q < 0.01 and *** q < 0.001). The 306 testable KEGG Level 3 pathways were treated as a separate multiple-testing family. Different lowercase letters indicate significant pairwise differences among treatments (*p* < 0.05).

## 3. Results

### 3.1. Endpoint Water-Quality Responses to Bellamya Stocking Density

**Figure 1:**
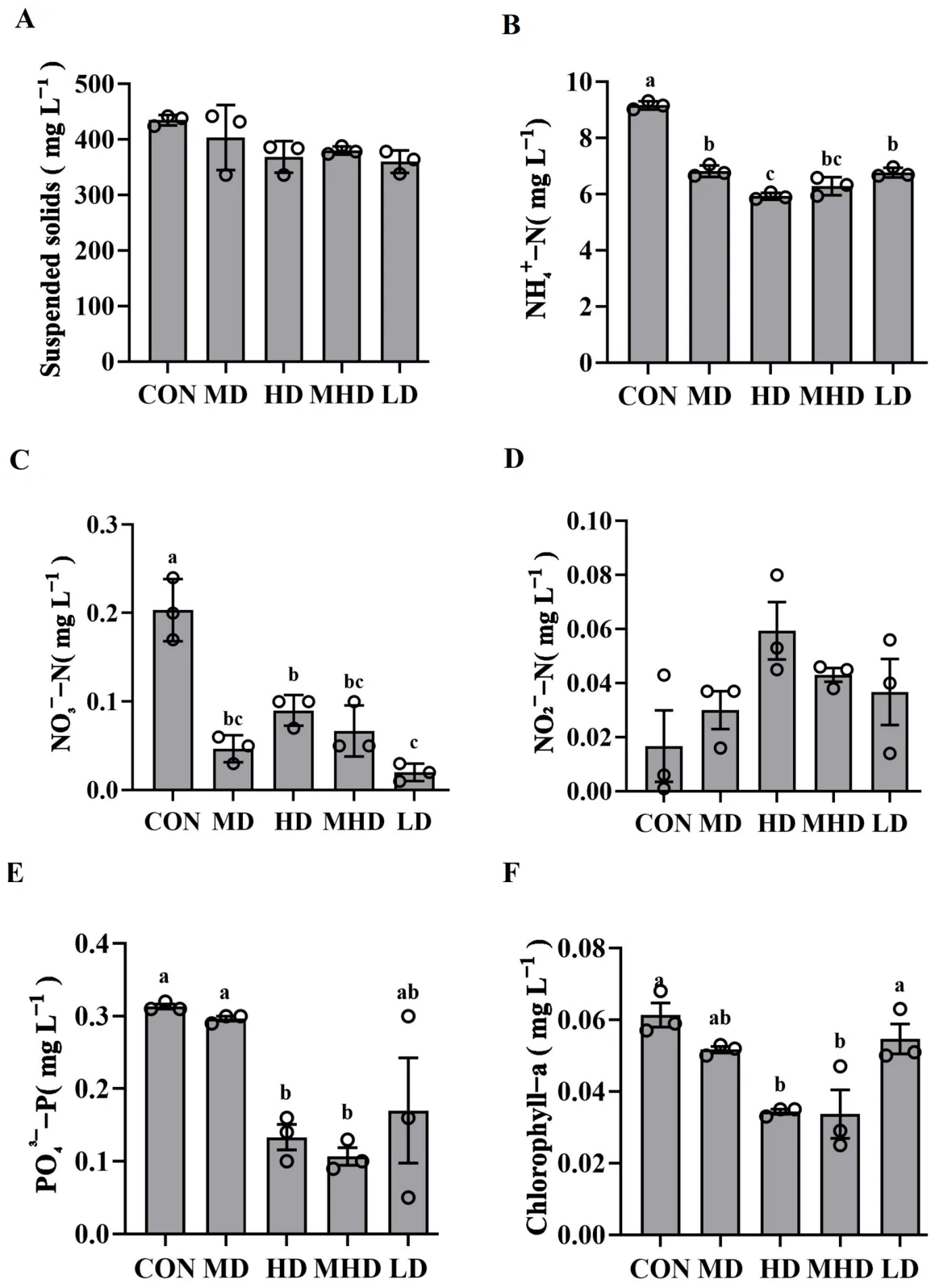
Effects of *Bellamya* stocking-density treatments on water-quality variables in aquaculture effluent. (**A**) Suspended solids (SS), (**B**) NH_4_^+^-N, (**C**) NO_3_^−^-N, (**D**) NO_2_^−^-N, (**E**) PO_4_^3−^-P, and (**F**) chlorophyll a (Chl-a). Each point represents an independent biological replicate (*n* = 3). Data are presented as mean ± SEM. Different lowercase letters indicate significant differences among treatments (*p* < 0.05). Treatment abbreviations are defined in Section 2.

At the 60-day endpoint, water-quality variables differed among *Bellamya* stocking-density treatments (Figure 1). SS showed a nonsignificant downward tendency across treatments (one-way ANOVA, F_4,10_ = 2.844, *p* = 0.082). NH_4_^+^-N was significantly lower in all *Bellamya*-stocked treatments than in CON, with the lowest concentration in HD. NO_3_^−^-N was also significantly lower in all stocked treatments, with the lowest concentration in LD. PO_4_^3−^-P was lower in HD and MHD than in CON, whereas LD showed greater among-replicate variation. Chl-a was significantly lower in HD and MHD. By contrast, NO_2_^−^-N showed a nonsignificant upward tendency, particularly in HD (one-way ANOVA, F_4,10_ = 2.530, *p* = 0.107). Thus, *Bellamya* stocking was associated with lower endpoint concentrations of several eutrophication-related variables, accompanied by a nonsignificant tendency toward higher nitrite.

### 3.2. Sequencing Coverage and Alpha Diversity

**Figure 2:**
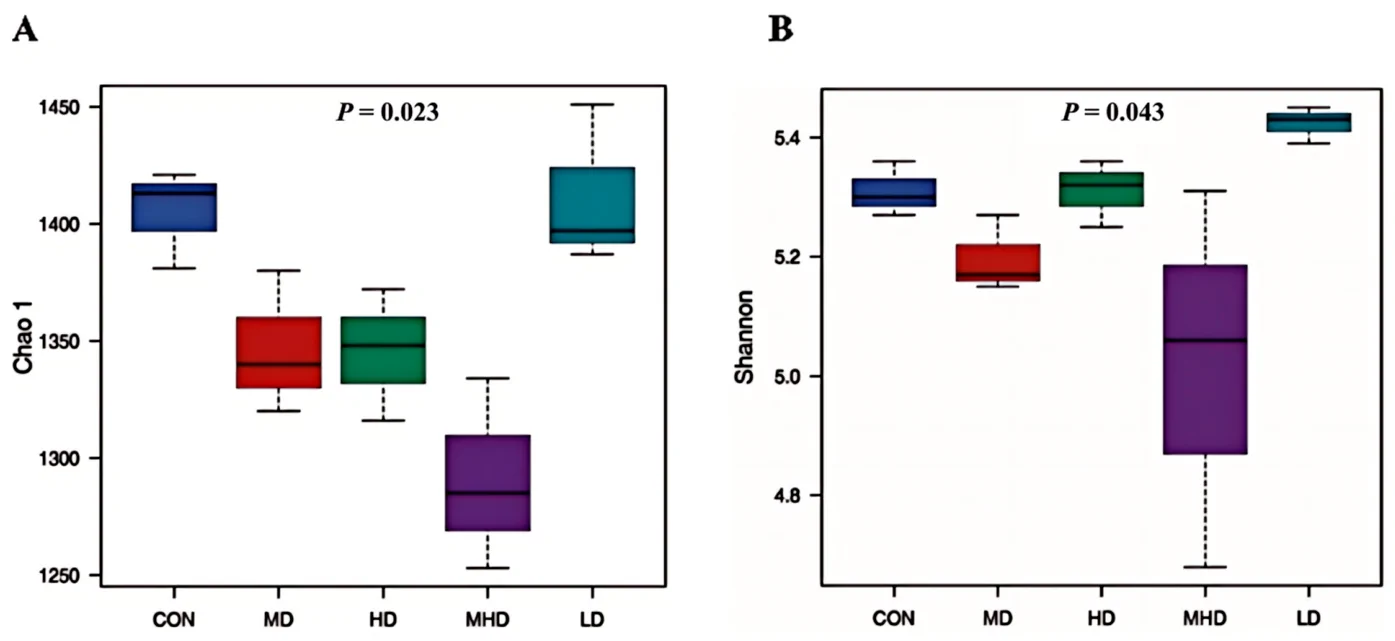
Alpha diversity of bacterial communities among treatments. (**A**) Chao1 richness and (**B**) Shannon diversity. Each boxplot represents three independent tank replicates (*n* = 3). The center line denotes the median, the box represents the interquartile range, and the whiskers show the observed range. Overall treatment effects were evaluated using the Kruskal–Wallis test; *p*-values are shown in the panels.

The 16S rRNA gene sequencing dataset contained 60,006–91,528 valid reads per sample and 1119–1334 observed OTUs. Good’s coverage was consistently high (99.64–99.80%), and rarefaction curves approached saturation, indicating adequate sequencing depth (Table S1; Figure S3). Chao1 richness differed among treatments (*p* = 0.023, Figure 2A), with lower values in MHD and higher values in LD and CON. An examination of the three MHD Chao1 values (1285, 1253 and 1334) did not identify a single sample that accounted for the lower treatment mean. Shannon diversity also differed among treatments (*p* = 0.043, Figure 2B); LD retained a relatively high diversity, whereas MHD showed the greatest among-replicate variation. This variation was mainly associated with the lower Shannon value in MHD1 (4.68) relative to MHD2 (5.06) and MHD3 (5.31). All MHD samples had 65,263–82,953 valid reads, a Good’s coverage of 99.68–99.77%, and saturated rarefaction profiles; therefore, no sample was excluded on the basis of sequencing-quality evidence.

### 3.3. Treatment-Associated Differences in Bacterial Community Structure

**Figure 3:**
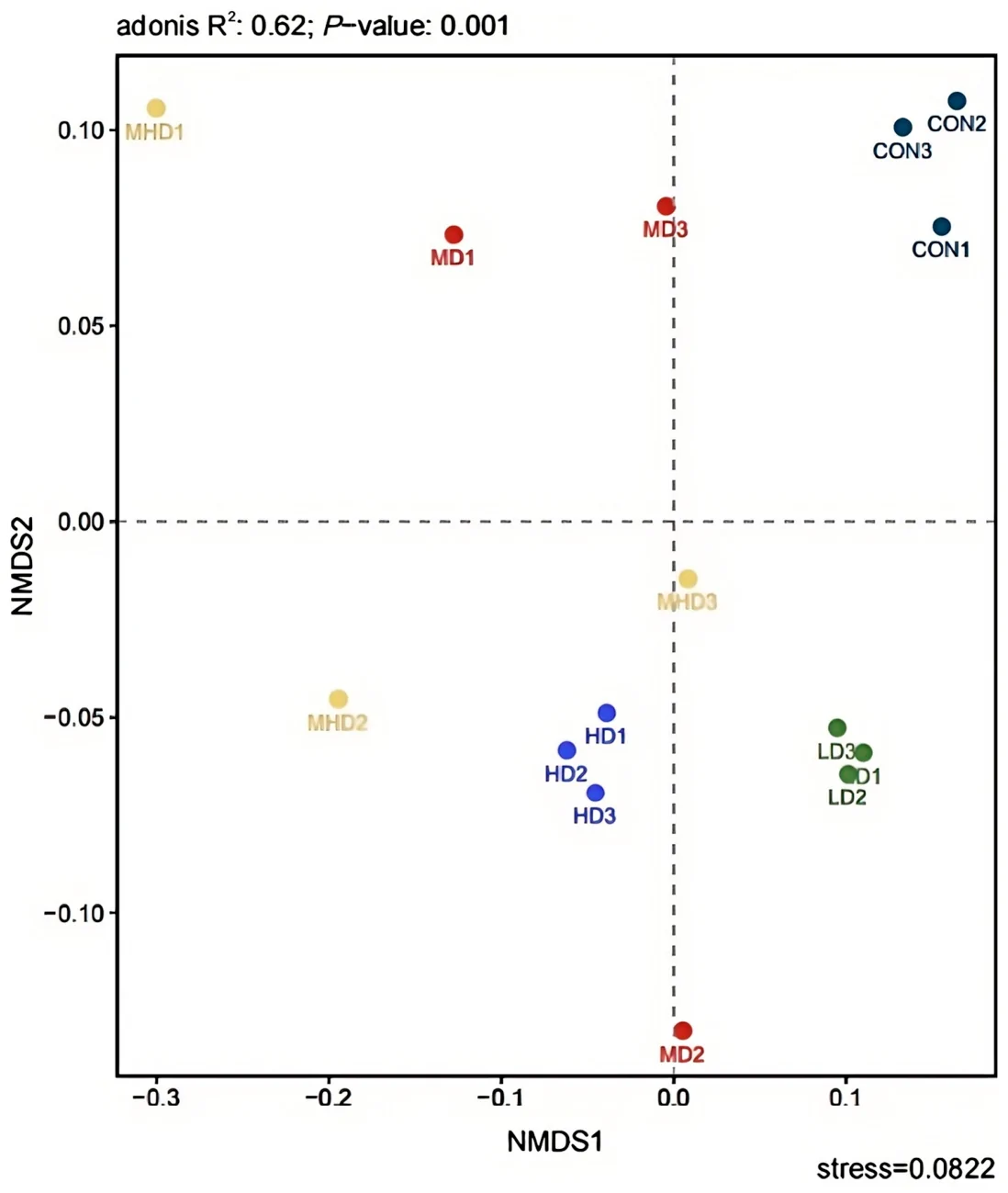
Non-metric multidimensional scaling (NMDS) ordination of bacterial communities based on Bray–Curtis dissimilarity calculated from the normalized OTU table. Each point represents an independent sample (*n* = 3 per treatment). PERMANOVA indicated treatment-associated differences (R^2^ = 0.62, *p* = 0.001; 999 permutations), and the final stress value was 0.0822. PERMDISP based on the same distance matrix was also significant (F_4,10_ = 4.99, *p* = 0.001), indicating unequal within-group dispersion; sample distances to treatment-group spatial medians are shown in Figure S4.

NMDS based on Bray–Curtis dissimilarity showed treatment-associated separation of bacterial communities (stress = 0.0822; Figure 3). PERMANOVA detected significant differences among treatments (R^2^ = 0.62, *p* = 0.001). PERMDISP based on the same normalized OTU table and distance matrix was also significant (F_4,10_ = 4.99, *p* = 0.001; Figure S4), with greater within-group dispersion in the MD and MHD treatments. Thus, the observed among-treatment differences were accompanied by heterogeneity in within-treatment dispersion.

### 3.4. Community Composition and Representative Bacterial Genera

**Figure 4:**
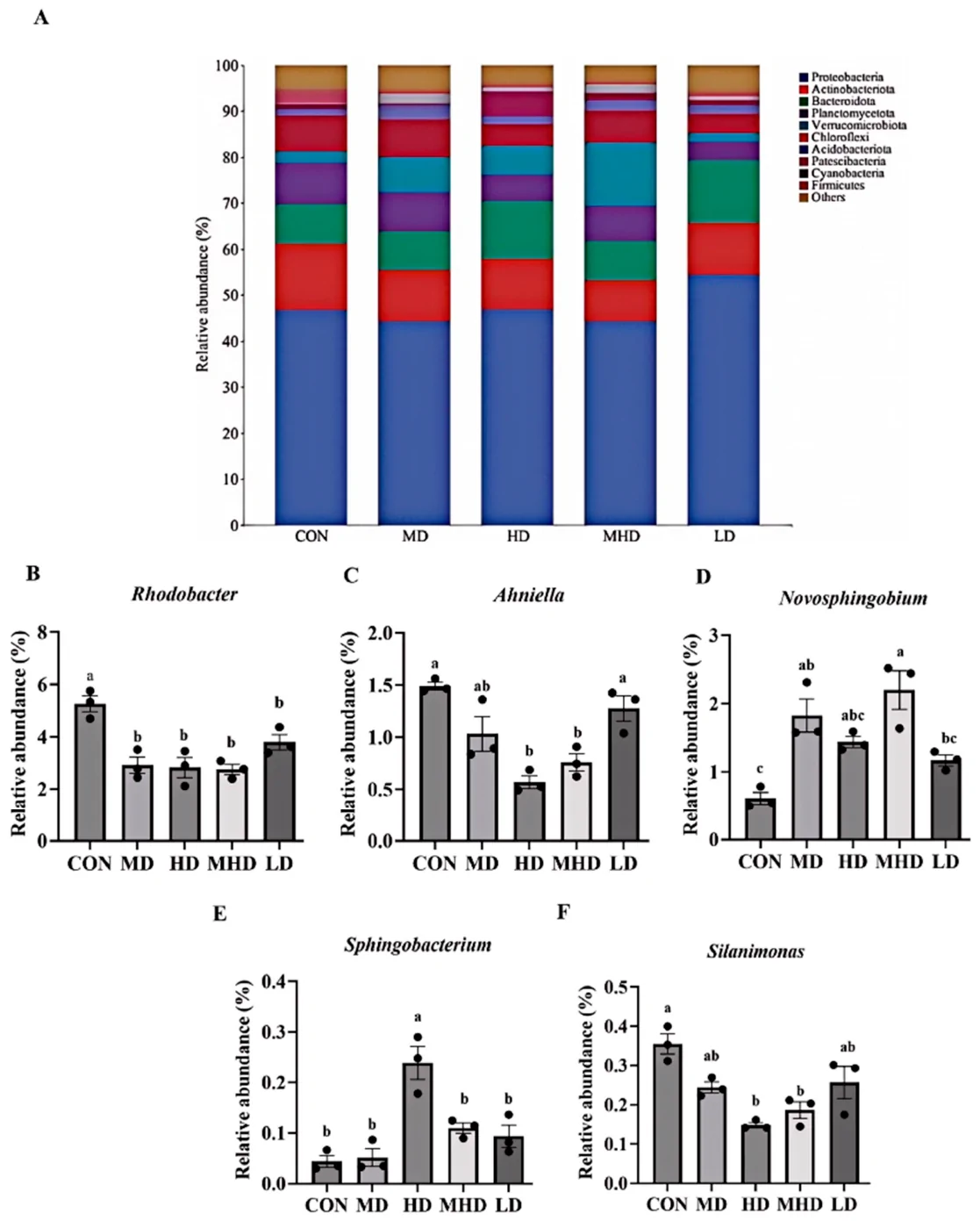
Bacterial community composition and relative abundances of representative genera. (**A**) Dominant bacterial phyla across treatments. (**B**–**F**) Sample-level relative abundances of *Rhodobacter*, *Ahniella*, *Novosphingobium*, *Sphingobacterium* and *Silanimonas*. These genera were identified among the LEfSe-discriminative taxa and selected as illustrative, nonexhaustive examples based on unambiguous genus-level annotation, sufficient abundance for visualization, and distinct, nonredundant treatment-associated patterns; all 25 discriminative taxa are listed in Supplementary Table S2. Points represent independent tank replicates (*n* = 3), bars show mean ± SEM, and different lowercase letters indicate significant pairwise differences (*p* < 0.05).

At the phylum level, the bacterial community was dominated by *Proteobacteria*, *Actinobacteriota*, *Bacteroidota*, *Planctomycetota*, *Verrucomicrobiota*, *Chloroflexi* and *Cyanobacteria*, with treatment-associated variation in relative abundance (Figure 4A). The top 20 genus profile contained a large “Others” fraction, demonstrating substantial contribution from numerous low-abundance genera (Figure S1).

Five named genera identified among the LEfSe-discriminative taxa were visualized as illustrative, nonexhaustive examples of genus-level treatment-associated abundance patterns (Figure 4B–F); the complete set of 25 discriminative taxa is provided in Table S2. *Rhodobacter* was more abundant in CON than in all *Bellamya* treatments. *Ahniella* was relatively enriched in CON and LD, whereas HD and MHD showed lower abundance. *Novosphingobium* increased in the treatment groups and reached its highest abundance in MHD, while CON had the lowest abundance. *Sphingobacterium* was selectively enriched in HD. *Silanimonas* was most abundant in CON and was lower in HD and MHD, with MD and LD showing intermediate values.

### 3.5. LEfSe Identified Treatment-Specific Discriminative Taxa

**Figure 5:**
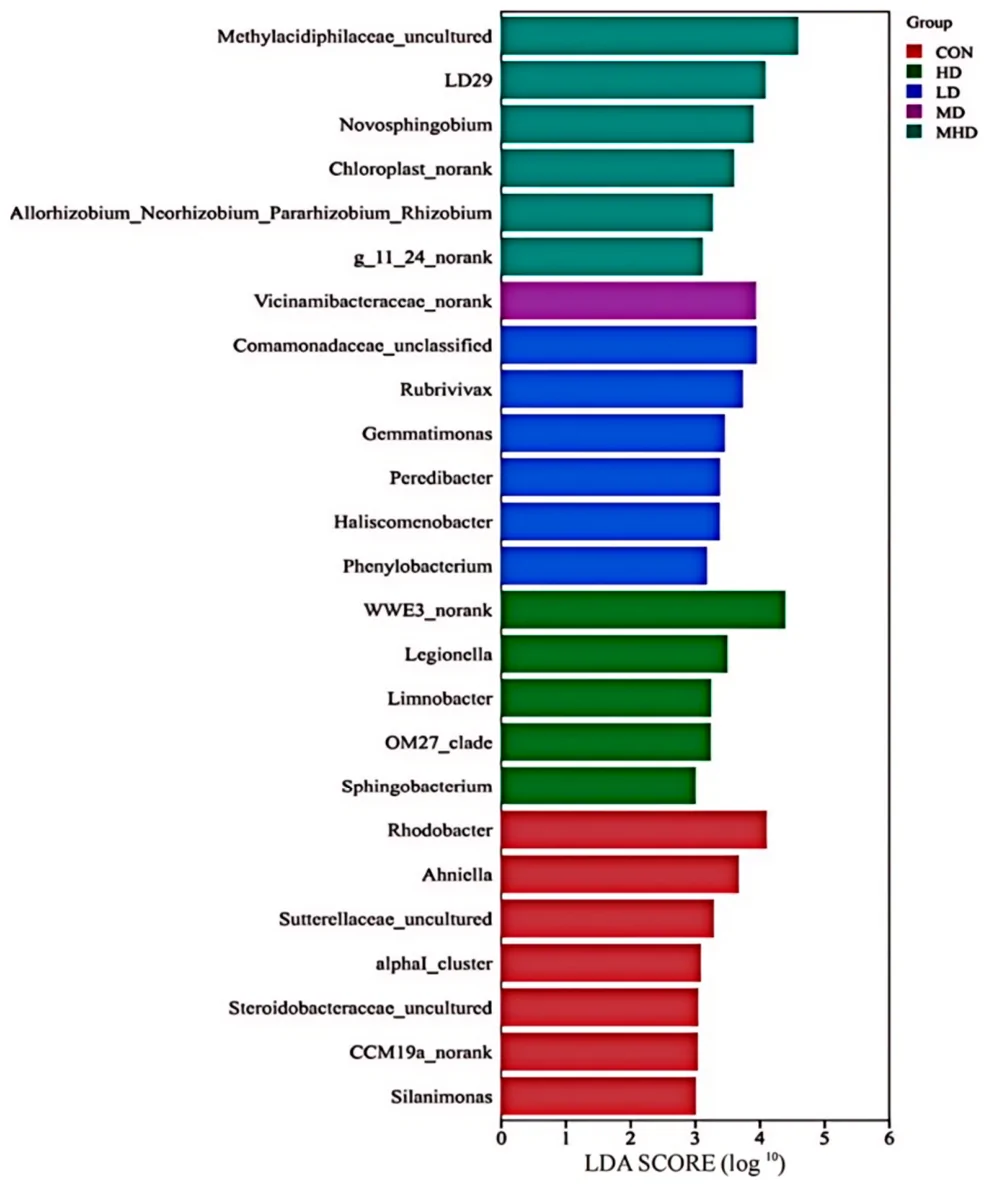
LEfSe-identified discriminative bacterial taxa among treatments. Twenty-five taxa meeting *p* < 0.05 and LDA score ≥ 3.0 are shown; bar colors denote the treatment in which each taxon was enriched. Because the output includes named genera together with no-rank, unclassified, uncultured and higher-level lineages, the results are reported as discriminative taxa rather than exclusively as genera.

LEfSe identified 25 discriminative taxa among the five groups using *p* < 0.05 and LDA score ≥ 3.0 (Figure 5; Table S2). CON was characterized by taxa including *Rhodobacter*, *Ahniella* and *Silanimonas*. MHD was characterized by *Novosphingobium* and several additional lineages, whereas HD was characterized by *Sphingobacterium* and other treatment-specific taxa. Because some discriminative features were annotated as no-rank, unclassified or uncultured lineages, the LEfSe results were interpreted at the level of discriminative taxa rather than exclusively as named genera.

### 3.6. Associations Between Selected Genera and Water-Quality Gradients

**Figure 6:**
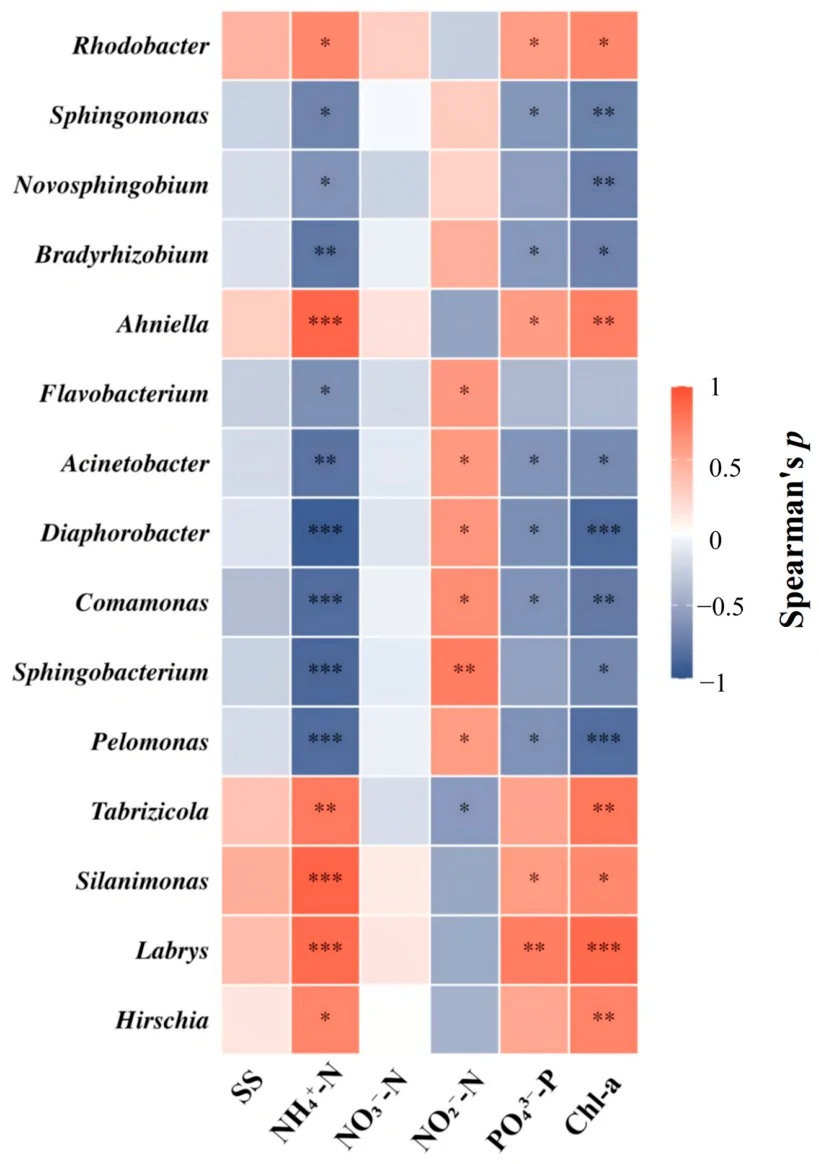
Spearman correlations between 15 selected bacterial genera and six water-quality variables (*n* = 15). Cell shading represents Spearman’s ρ according to the color scale. Asterisks denote Benjamini–Hochberg-adjusted significance across all 90 tests (* q < 0.05, ** q < 0.01 and *** q < 0.001).

**Figure 7:**
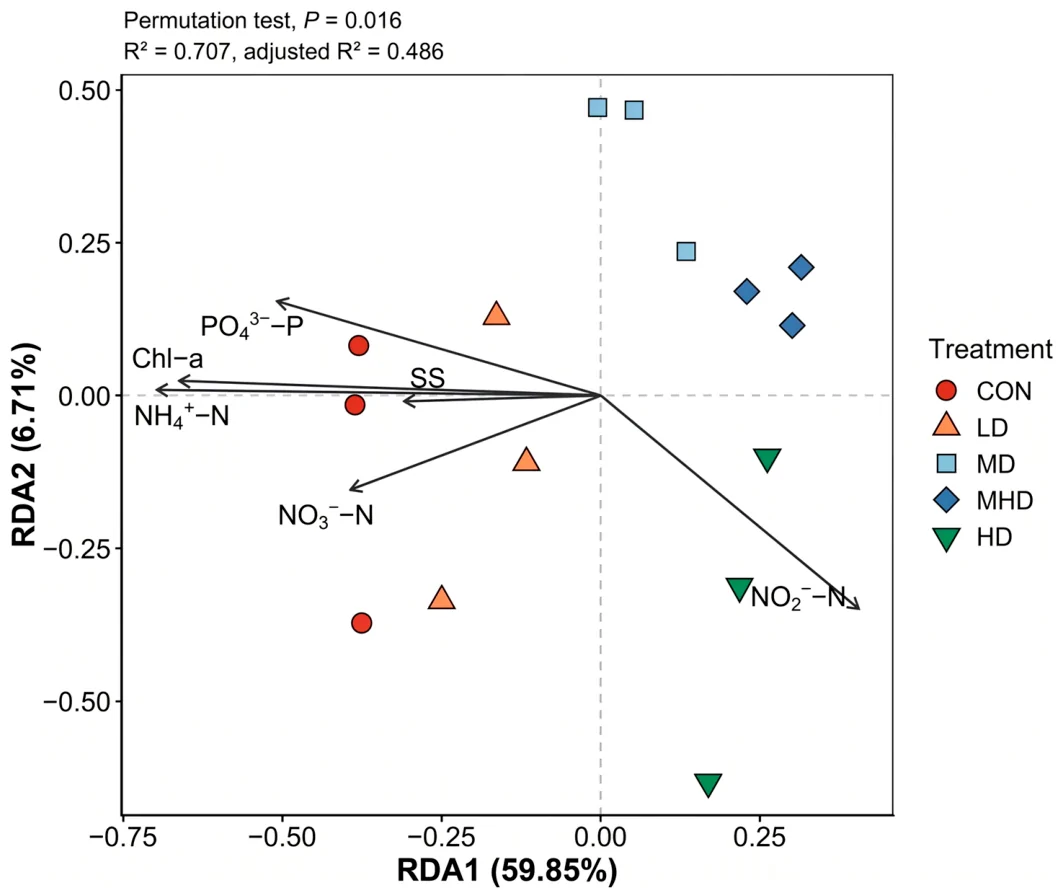
Hellinger-based redundancy analysis (RDA) relating the 15 selected bacterial genera to six water-quality variables. Genus-level relative abundances were Hellinger transformed and environmental variables were z-score standardized. Overall and axis-wise significance was assessed using 999 permutations. The overall model was significant (*p* = 0.016; R^2^ = 0.7065; adjusted R^2^ = 0.4864). RDA1 explained 59.85% of total variation and was significant (*p* = 0.014), whereas RDA2 explained 6.71% and was not significant (*p* = 0.761). None of the six environmental variables showed a significant independent marginal effect after Benjamini–Hochberg correction (all q > 0.05).

Spearman analysis identified structured genus–environment associations, particularly for NH_4_^+^-N and Chl-a (Figure 6). Genera including *Sphingomonas*, *Novosphingobium*, *Bradyrhizobium*, *Acinetobacter*, *Diaphorobacter*, *Comamonas*, *Sphingobacterium* and *Pelomonas* were generally negatively associated with NH_4_^+^-N and Chl-a, whereas *Rhodobacter*, *Ahniella*, *Silanimonas*, *Tabrizicola*, *Labrys* and *Hirschia* showed the opposite pattern. Several treatment-associated genera were positively related to NO_2_^−^-N. After joint Benjamini–Hochberg correction of all 90 tests, 46 genus–environment associations remained significant (Figure 6; Table S3). The Hellinger-based RDA demonstrated a significant overall multivariate association between the selected genera and the six measured water-quality variables (999-permutation test, *p* = 0.016; R^2^ = 0.7065; adjusted R^2^ = 0.4864; Figure 7; Table S5). RDA1 and RDA2 accounted for 59.85% and 6.71% of the total variation, respectively. RDA1 was significant (*p* = 0.014), whereas RDA2 was not significant (*p* = 0.761). None of the six environmental variables showed a significant independent marginal effect after Benjamini–Hochberg correction (all q > 0.05). VIFs indicated strong collinearity for NH_4_^+^-N (11.960) and elevated collinearity for NO_3_^−^-N (5.853) and Chl-a (7.589). NH_4_^+^-N, PO_4_^3−^-P and Chl-a were oriented toward the ordination space occupied primarily by CON samples, whereas NO_2_^−^-N showed an opposing orientation and aligned more closely with several *Bellamya*-stocked samples.

### 3.7. Predicted KEGG Functional Trends

At KEGG Level 2, predicted functions were dominated by metabolism, membrane transport, genetic-information processing, energy metabolism, and carbohydrate- and amino-acid-related categories (Figure S2). At Level 3, 328 pathways were available; 22 all-zero or invariant pathways were excluded and 306 were tested. Fifty-nine pathways had raw *p* < 0.05, but none remained significant after Benjamini–Hochberg correction (all q ≥ 0.05; Supplementary Table S6). The focused panel of 16 environmentally relevant pathways displayed in Figure S5 therefore represents descriptive predicted functional trends rather than statistically supported pathway activation. Complete pathway-level statistics are provided in Supplementary Data S1.

## 4. Discussion

### 4.1. Bellamya Stocking Density Was Associated with Lower Endpoint Nutrient and Algal-Biomass Indicators

At the sampling endpoint, *Bellamya*-stocked treatments had lower NH_4_^+^-N and NO_3_^−^-N concentrations than CON, and selected densities also had lower PO_4_^3−^-P and Chl-a. These findings indicate potential for *Bellamya* as a low-input biological component of aquaculture-effluent management. Relative to treatment systems requiring continuous aeration or chemical addition, gastropod-mediated treatment may influence several endpoints through grazing, particle ingestion, biodeposition and modification of microbial habitat [2,3,4,5,11,12,13,14,15,16,17].

The present design cannot separate grazing, biodeposition, excretion and microbial habitat modification, so these processes remain plausible explanations rather than demonstrated mechanisms. The absence of a significant SS response (*p* = 0.082), despite a numerical decline, also shows that treatment effects were endpoint-specific and variable among the three replicate tanks.

The significant endpoint differences in dissolved nutrients and Chl-a provide stronger evidence than the nonsignificant SS tendency; however, no single endpoint establishes a complete treatment mechanism.

The HD biomass density was 24.31 kg m^−3^, approximately 4.0 times LD, 2.6 times MD and 1.9 times MHD. Although HD and MHD produced relatively strong responses for several endpoints, the experiment did not include an economic assessment nor establish an optimal commercial density. Lower densities combined with longer treatment or hydraulic-retention periods may offer a better cost–benefit balance and require field-scale testing.

### 4.2. Nitrite Accumulation Suggests an Imbalance Among Nitrogen-Transformation Processes

NO_2_^−^-N exhibited a different response from NH_4_^+^-N and NO_3_^−^-N. Although the overall treatment effect was not significant, several *Bellamya*-stocked treatments had numerically higher nitrite concentrations than CON. Nitrite is an intermediate shared by nitrification, denitrification and other nitrogen-transformation processes, and it can accumulate when production and consumption rates are temporarily imbalanced [6,7,8]. The endpoint pattern therefore does not negate the lower ammonia and nitrate concentrations; rather, it suggests that the balance among nitrogen-transformation processes may not have reached a steady state by the time of sampling.

Recent work on *Bellamya purificata* also indicates that snail density and bioturbation can alter nitrogen forms and bacterial communities in ways that differ among nitrogen pools [14,15]. In those systems, changes in dissolved oxygen, nitrate and nitrite were closely related to shifts in bacterial community composition [15]. The current result is compatible with such asynchronous responses, but direct attribution to nitrification or denitrification would require functional-gene or process-rate measurements. A conservative interpretation is therefore that *Bellamya* treatment modified the conditions under which nitrogen transformations occurred, producing lower ammonia and nitrate together with a possible transient imbalance between nitrite production and consumption.

The maximum NO_2_^−^-N concentration measured in the present experiment was 0.080 mg L^−1^. This value was below the 0.44 mg L^−1^ NO_2_^−^-N exposure level at which chronic nitrite accumulation in plasma and muscle was reported in juvenile pike-perch, but nitrite toxicity varies substantially among cultured species and is strongly modified by chloride concentration, temperature, dissolved oxygen, exposure duration, body size and age [46,47]. The observed concentration should therefore not be regarded as universally safe. Species-specific risk assessment, together with chloride and oxygen measurements, would be required before the treated effluent is reused for culture or discharged to sensitive receiving waters.

### 4.3. Treatment-Associated Bacterial Community Differences and Dispersion

NMDS and PERMANOVA indicated treatment-associated differences in bacterial communities, but the significant PERMDISP result showed that multivariate dispersion differed among treatments. In particular, MD and MHD were more dispersed than the other groups. Consequently, the PERMANOVA result cannot be attributed solely to shifts in group centroids; treatment-related differences in both centroid position and within-treatment dispersion likely contributed to the observed separation.

The dominant phyla were typical of freshwater and aquaculture environments, while the large genus-level “Others” fraction indicated that numerous low-abundance taxa contributed collectively to community structure. This justified retaining the top 20 genus profile as supplementary context rather than treating dominant genera alone as a complete description of the community [9,32,48].

The overall pattern is consistent with previous *Bellamya*-associated studies showing changes in sediment and host-associated microbial communities [15,17,18], although the direction and magnitude of the response depend on species, habitat compartment and stocking level.

### 4.4. Associations of Representative Genera with Water-Quality Gradients

The five displayed genera were illustrative, nonexhaustive examples of LEfSe-supported treatment-associated patterns. Their abundance distributions and correlations with nutrients and Chl-a gradients support their use as candidate treatment indicators, but do not demonstrate direct pollutant removal or a causal role in effluent treatment.

Several genera that increased in *Bellamya*-stocked treatments were negatively associated with NH_4_^+^-N and Chl-a and positively associated with NO_2_^−^-N. Reported denitrification or other nitrogen-related capacities in selected *Comamonas* and *Bradyrhizobium* strains provide ecological context [49,50,51,52]; however, genus-level 16S assignments encompass substantial functional heterogeneity. These associations therefore support hypothesis generation rather than the inference of specific metabolic pathways.

The RDA supported a shared multivariable association between the selected genera and the combined water-quality gradient. NH_4_^+^-N, PO_4_^3−^-P and Chl-a were oriented toward the control-associated community, whereas NO_2_^−^-N showed an opposing gradient. Because shared treatment responses can generate correlations without direct causality, the selected taxa are described as associated or treatment-responsive rather than as drivers or mediators. The absence of significant independent marginal effects, together with elevated VIFs for NH_4_^+^-N, NO_3_^−^-N and Chl-a, further indicates that the ordination reflects shared environmental gradients rather than a single dominant predictor.

A host-source contribution cannot be excluded. The gut, shell surface and mucus microbiota of the experimental snails were not sequenced, so the current design cannot distinguish bacteria released or shed by *Bellamya* from resident water-column taxa that responded to nutrient and habitat changes. Previous studies demonstrate that *Bellamya* gut communities can differ from surrounding epiphytic or environmental communities and can respond to environmental conditions [18,19]. Accordingly, *Rhodobacter*, *Ahniella*, *Silanimonas*, *Novosphingobium* and *Sphingobacterium* are interpreted here as treatment-associated water-column indicators rather than as unequivocal environmental responders or taxa shown to have arisen solely through in situ environmental selection.

### 4.5. Functional Inference, Study Limitations and Application Perspective

Predicted functions were dominated by broad metabolic and transport categories. Although 59 Level 3 pathways had raw *p* < 0.05, none of the 306 testable pathways remained significant after false-discovery-rate correction (Supplementary Table S6; Supplementary Data S1). The Level 3 heatmap therefore summarizes descriptive functional predictions and does not support claims of pathway activation. This conservative interpretation is also warranted because 16S-based functional inference is indirect and taxonomic turnover does not necessarily imply equivalent functional turnover [29,30,31,32].

The present study has several limitations. First, each treatment contained three independent tanks, limiting statistical power and the precision of treatment-effect estimates. Second, sampling was conducted at a single endpoint, so temporal succession in water quality and bacterial communities could not be resolved. Third, nitrogen-cycling genes, enzyme activities and process rates were not measured directly; consequently, the microbiome data support ecological associations rather than a complete mechanistic pathway. Fourth, the relative contributions of grazing, biodeposition, excretion and microbial-habitat modification were not separated experimentally. Fifth, no extraction blank was included in the original DNA-extraction workflow, whereas a batch-level no-template PCR control (CK) was included in the routine PCR quality-control workflow and showed no visible amplicon band on agarose gel electrophoresis. Because the CK control was not sequenced, potential extraction-stage contaminants and low-level sequences below the gel-detection threshold could not be evaluated directly. This limitation is most relevant to rare and low-abundance taxa; accordingly, interpretation emphasized community-level patterns, prevalent taxa and treatment-associated responses reproduced across biological replicates.

Despite these limitations, the study identified lower endpoint concentrations of several water-quality variables, treatment-associated differences in bacterial communities and reproducible associations between selected genera and environmental gradients. Unequal multivariate dispersion, the absence of direct functional measurements and the experimental limitations described above constrain mechanistic inference but do not preclude the evaluation of *Bellamya* as a candidate ecological component of aquaculture effluent management. Long-term stability, field performance and process-level mechanisms require further investigation.

## 5. Conclusions

At the 60-day endpoint, *Bellamya*-stocked treatments had lower NH_4_^+^-N and NO_3_^−^-N concentrations than CON, and selected densities also had lower PO_4_^3−^-P and Chl-a, whereas NO_2_^−^-N showed a nonsignificant upward tendency. Alpha diversity differed among treatments, and bacterial communities showed treatment-associated differences; however, significant heterogeneity in multivariate dispersion requires cautious interpretation of the PERMANOVA result. Representative taxa and the predefined 15-genus panel were associated with nutrient and algal-biomass gradients, but these relationships do not establish causality. No predicted KEGG Level 3 pathway remained significant after false-discovery-rate correction. Collectively, the findings support further evaluation of *Bellamya* stocking as an ecological component of aquaculture-effluent management; however, the microbiome data indicate community-level associations and predicted functional trends rather than a confirmed microbial mechanism.

## Figures and Tables

**Table 1:** Experimental treatments and initial *Bellamya* stocking densities.

Parameter	CON	LD	MD	MHD	HD
Number of *Bellamya* (ind. tank^−1^)	0	15	25	30	65
Initial total wet weight (g tank^−1^)	0	62.35 ± 0.81	96.36 ± 1.05	132.25 ± 1.84	247.94 ± 1.03
Water volume (L tank^−1^)	10.2	10.2	10.2	10.2	10.2
Biomass stocking density (kg m^−3^)	0	6.11 ± 0.08	9.45 ± 0.10	12.97 ± 0.18	24.31 ± 0.10
Numerical stocking density (ind. L^−1^)	0	1.47	2.45	2.94	6.37
Calculated mean wet weight per stocked individual (g ind.^−1^)	0	4.16 ± 0.05	3.85 ± 0.04	4.41 ± 0.06	3.81 ± 0.02

Note: Numerical stocking density was calculated as stocking number ÷ 10.2 L. Calculated mean wet weight per stocked individual was calculated as group mean initial total wet weight per tank ÷ stocking number; the accompanying ± value was scaled by the same divisor. These derived values summarize group-level stocking mass and do not represent directly measured variation among individual snails.

## Data Availability

The original contributions presented in this study are included in the article and Supplementary Material. Further inquiries can be directed to the corresponding author.

## Supplementary Materials

The following supporting information can be downloaded at: https://www.mdpi.com/article/10.3390/microorganisms14081813/s1, Table S1. Sequencing depth and alpha-diversity indices of bacterial communities in aquaculture tailwater samples, Table S2. Differentially enriched bacterial taxa identified by LEfSe analysis. Table S3. Spearman correlation coefficients and Benjamini–Hochberg-adjusted q values for 15 selected genera and six water-quality variables. Table S4. Evidence supporting the inclusion of the 15 genera in the targeted taxon–environment analyses. Table S5. Permutation tests and collinearity diagnostics for the Hellinger-based redundancy analysis (RDA).Table S6. Summary of the statistical analysis of PICRUSt2-predicted KEGG Level 3 pathways. Figure S1. Genus-level bacterial community composition among Bellamya stocking-density treatments. Bars show treatment-level mean relative abundances of the 20 most abundant genus-level taxa in the normalized OTU table; all remaining taxa were grouped as “Others”. Figure S2. PICRUSt2-predicted KEGG Level 2 functional composition. Bars show the mean within-sample relative abundance of predicted functional categories across the 15 samples. These values are 16S-based predictions and do not represent direct measurements of genes, transcription, enzyme activity or pathway flux. Figure S3. Rarefaction curves for bacterial OTUs clustered at 97% sequence similarity. Each curve represents one sample; the x-axis indicates sequencing depth and the y-axis observed OTUs. Curves approaching a plateau indicate adequate sequencing coverage. Figure S4. Multivariate dispersion of bacterial communities based on Bray–Curtis dissimilarity. Boxplots show sample distances to the treatment-group spatial median (*n* = 3 per treatment), calculated using betadisper (type = “median”); larger values indicate greater within-group dispersion. PERMDISP: F_4,10_ = 4.99, P = 0.001 (999 permutations). Figure S5. PICRUSt2-predicted KEGG Level 3 functional trends among Bellamya stocking-density treatments. Pathway abundances were converted to within-sample relative abundances, treatment means were calculated, and rows were standardized as Z-scores. The right-hand values are Benjamini–Hochberg-adjusted q values calculated across all 306 testable pathways. No pathway remained significant after FDR correction (all q ≥ 0.05); the heatmap is therefore descriptive and does not demonstrate pathway activation or inhibition. Supplementary Data S1: Complete pathway-level statistics.
