# Supplementary material for: Effects of Bellamya Stocking Density on Water Quality and Bacterial Community Responses in Aquaculture Effluent
Source: Microorganisms. 2026 Aug 17;14(8):1813. doi: 10.3390/microorganisms14081813 (PMC13515823; doi:10.3390/microorganisms14081813)
Supplement: Supplementary file 1 [file microorganisms-14-01813-s001.zip › Ring screw supplementary table Table S1.pdf]

Table S1. Sequencing depth and alpha-diversity indices of bacterial communities in aquaculture tailwater samples.

Sample ID	Group	Valid reads	OTUs (97% similarity)	ACE (95% CI)	Chao1 (95% CI)	Good's coverage (%)	Shannon (95% CI)	Simpson (95% CI)
CON1	CON	67,427	1271	1418 (1383-1464)	1413 (1372-1472)	99.68	5.3 (5.29-5.31)	0.0125 (0.0123-0.0128)
CON2	CON	66,903	1249	1398 (1363-1445)	1381 (1343-1436)	99.67	5.27 (5.25-5.28)	0.0123 (0.0121-0.0125)
CON3	CON	63,680	1265	1429 (1391-1478)	1421 (1377-1484)	99.64	5.36 (5.34-5.37)	0.011 (0.0108-0.0112)
HD1	HD	67,460	1222	1372 (1336-1420)	1372 (1327-1434)	99.69	5.36 (5.34-5.37)	0.0109 (0.0108-0.0111)
HD2	HD	60,006	1174	1315 (1281-1360)	1316 (1274-1376)	99.65	5.25 (5.24-5.26)	0.0129 (0.0127-0.0132)
HD3	HD	67,957	1186	1365 (1323-1419)	1348 (1301-1413)	99.67	5.32 (5.3-5.33)	0.0117 (0.0115-0.0119)
LD1	LD	90,588	1334	1470 (1437-1513)	1451 (1416-1502)	99.78	5.45 (5.44-5.46)	0.0094 (0.0093-0.0095)
LD2	LD	80,088	1257	1393 (1360-1438)	1387 (1348-1443)	99.75	5.43 (5.42-5.43)	0.0098 (0.0096-0.0099)
LD3	LD	91,528	1286	1410 (1379-1451)	1397 (1362-1446)	99.79	5.39 (5.38-5.4)	0.0104 (0.0102-0.0105)
MD1	MD	89,464	1249	1383 (1350-1427)	1380 (1340-1437)	99.78	5.15 (5.14-5.16)	0.016 (0.0158-0.0163)
MD2	MD	90,879	1213	1338 (1307-1380)	1320 (1287-1369)	99.80	5.27 (5.26-5.28)	0.0118 (0.0116-0.0119)
MD3	MD	67,283	1133	1319 (1276-1376)	1340 (1281-1422)	99.65	5.17 (5.15-5.18)	0.013 (0.0128-0.0132)
MHD1	MHD	75,978	1136	1300 (1261-1351)	1285 (1242-1347)	99.72	4.68 (4.67-4.7)	0.0385 (0.0377-0.0393)
MHD2	MHD	65,263	1119	1281 (1242-1331)	1253 (1213-1309)	99.68	5.06 (5.05-5.07)	0.0159 (0.0156-0.0162)
MHD3	MHD	82,953	1151	1278 (1245-1322)	1334 (1277-1417)	99.77	5.31 (5.3-5.33)	0.0103 (0.0102-0.0105)

Note. Valid reads are quality-filtered reads before rarefaction. OTUs were clustered at 97% sequence similarity. Values in parentheses are 95% confidence intervals, and Good’s coverage is expressed as a percentage. Alpha-diversity indices and rarefaction curves were taken from the provider-generated 97%-similarity OTU output. The normalized OTU table used for downstream community analyses was rarefied to 60,006 reads per sample.

Table S2. Differentially enriched bacterial taxa identified by LEfSe analysis.

Taxon	Group	LDA_score	P-value
Rhodobacter	CON	4.09674	0.0421463
Ahniella	CON	3.66638	0.0148231
Sutterellaceae_uncultured	CON	3.2821	0.0337278
alphaI_cluster	CON	3.07951	0.0137961
Steroidobacteraceae_uncultured	CON	3.0423	0.015551
CCM19a_norank	CON	3.03707	0.0156979
Silanimonas	CON	3.00023	0.0195518
Vicinamibacteraceae_norank	MD	3.93419	0.0148231
WWE3_norank	HD	4.39091	0.0433307
Legionella	HD	3.49492	0.0202967
Limnobacter	HD	3.24206	0.0377119
OM27_clade	HD	3.23511	0.0261919
Sphingobacterium	HD	3.00148	0.0277762
Methylacidiphilaceae_uncultured	MHD	4.59095	0.0261919
LD29	MHD	4.08531	0.0421463
Novosphingobium	MHD	3.89723	0.0121305
Chloroplast_norank	MHD	3.59798	0.0152544
Allorhizobium_Neorhizobium_Pararhizobium_Rhizobium	MHD	3.27044	0.0197273
g_11_24_norank	MHD	3.11104	0.0290782
Comamonadaceae_unclassified	LD	3.9485	0.0382406
Rubrivivax	LD	3.73537	0.0453106
Gemmatimonas	LD	3.4553	0.0366754
Peredibacter	LD	3.37969	0.0323409
Haliscomenobacter	LD	3.37422	0.0382406
Phenylobacterium	LD	3.17883	0.0258249

Note. Taxa satisfied Kruskal–Wallis P < 0.05 and LDA score ≥ 3.0. P values are unadjusted. “Enriched group” identifies the treatment associated with the highest discriminative abundance. The output includes named and incompletely classified taxa.

Table S3. Spearman correlation coefficients and Benjamini–Hochberg-adjusted q values for 15 selected genera and six water-quality variables.

Genus	water -quality variables					
	**SS**	**NH_4^+-N**	**NO_3^--N**	**NO_2^--N**	**PO_4^{3-}-P**	**Chl-a**
Rhodobacter	0.46 (0.140)	0.70* (0.012)	0.29 (0.414)	-0.28 (0.424)	0.59* (0.041)	0.71* (0.011)
Sphingomonas	-0.25 (0.463)	-0.71* (0.011)	-0.05 (0.858)	0.30 (0.391)	-0.60* (0.040)	-0.73** (0.009)
Novosphingobium	-0.20 (0.575)	-0.62* (0.033)	-0.25 (0.473)	0.26 (0.448)	-0.56 (0.057)	-0.75** (0.007)
Bradyrhizobium	-0.18 (0.616)	-0.79** (0.004)	-0.09 (0.771)	0.48 (0.115)	-0.59* (0.041)	-0.71* (0.011)
Ahniella	0.29 (0.416)	0.88*** (<0.001)	0.17 (0.635)	-0.53 (0.076)	0.59* (0.042)	0.75** (0.007)
Flavobacterium	-0.28 (0.430)	-0.64* (0.027)	-0.20 (0.575)	0.62* (0.034)	-0.41 (0.204)	-0.39 (0.227)
Acinetobacter	-0.21 (0.567)	-0.81** (0.002)	-0.13 (0.695)	0.61* (0.037)	-0.62* (0.034)	-0.67* (0.020)
Diaphorobacter	-0.16 (0.635)	-0.93*** (<0.001)	-0.15 (0.659)	0.63* (0.033)	-0.65* (0.026)	-0.86*** (<0.001)
Comamonas	-0.38 (0.235)	-0.85*** (<0.001)	-0.09 (0.772)	0.67* (0.018)	-0.63* (0.033)	-0.77** (0.005)
Sphingobacterium	-0.26 (0.448)	-0.87*** (<0.001)	-0.12 (0.722)	0.76** (0.005)	-0.54 (0.066)	-0.68* (0.017)
Pelomonas	-0.20 (0.575)	-0.84*** (<0.001)	-0.09 (0.771)	0.59* (0.041)	-0.63* (0.032)	-0.84*** (<0.001)
Tabrizicola	0.36 (0.279)	0.78** (0.004)	-0.19 (0.592)	-0.58* (0.044)	0.54 (0.066)	0.79** (0.003)
Silanimonas	0.49 (0.108)	0.89*** (<0.001)	0.12 (0.722)	-0.51 (0.091)	0.59* (0.041)	0.70* (0.013)
Labrys	0.39 (0.228)	0.85*** (<0.001)	0.15 (0.660)	-0.49 (0.107)	0.76** (0.005)	0.86*** (<0.001)
Hirschia	0.15 (0.660)	0.72* (0.010)	0.00 (0.990)	-0.44 (0.157)	0.52 (0.079)	0.73** (0.009)

Note. Entries are Spearman's ρ, with Benjamini–Hochberg-adjusted q values in parentheses. All 90 tests (15 genera × six variables) were adjusted jointly. Asterisks indicate *q < 0.05, **q < 0.01 and ***q < 0.001.

Table S4. Evidence supporting the inclusion of the 15 genera in the targeted taxon–environment analyses.

Genus	Overall abundance		Mean relative abundance by treatment (%)					Highest-mean group	Max/min fold	Kruskal–Wallis P	BH-adjusted q	LEfSe support
	Mean (%)	Prevalence(n/15)	CON	LD	MD	MHD	HD					
Rhodobacter	3.512	15/15	5.266	3.796	2.916	2.754	2.829	CON	1.91	0.042	0.043	Yes
Sphingomonas	1.903	15/15	0.695	0.818	2.110	3.569	2.324	MHD	5.14	0.025	0.036	No
Novosphingobium	1.450	15/15	0.612	1.168	1.829	2.202	1.439	MHD	3.60	0.012	0.036	Yes
Bradyrhizobium	1.017	15/15	0.606	0.703	0.957	1.557	1.260	MHD	2.57	0.024	0.036	No
Ahniella	1.026	15/15	1.492	1.277	1.032	0.760	0.570	CON	2.62	0.015	0.036	Yes
Flavobacterium	1.132	15/15	0.844	1.354	0.788	1.057	1.616	HD	2.05	0.029	0.036	No
Acinetobacter	0.186	15/15	0.066	0.186	0.140	0.255	0.286	HD	4.36	0.043	0.043	No
Diaphorobacter	0.169	15/15	0.037	0.110	0.103	0.232	0.361	HD	9.70	0.019	0.036	No
Comamonas	0.141	15/15	0.042	0.079	0.061	0.287	0.236	MHD	6.80	0.024	0.036	No
Sphingobacterium	0.108	15/15	0.044	0.094	0.052	0.110	0.239	HD	5.44	0.028	0.036	Yes
Pelomonas	0.172	15/15	0.091	0.123	0.160	0.266	0.220	MHD	2.93	0.012	0.036	No
Tabrizicola	0.127	15/15	0.194	0.186	0.143	0.062	0.052	CON	3.75	0.032	0.037	No
Silanimonas	0.238	15/15	0.355	0.257	0.244	0.187	0.148	CON	2.39	0.020	0.036	Yes
Labrys	0.230	15/15	0.311	0.253	0.229	0.170	0.187	CON	1.83	0.022	0.036	No
Hirschia	0.664	15/15	0.951	0.902	0.575	0.401	0.494	CON	2.37	0.021	0.036	No

Note. Abundance and prevalence were calculated across 15 samples. Max/min fold is the highest treatment mean divided by the lowest non-zero treatment mean. Kruskal–Wallis P values were Benjamini–Hochberg adjusted across the fixed 15-genus panel. LEfSe support indicates inclusion among the 25 discriminative taxa in Table S2. This exploratory panel does not imply functional or causal importance.

Table S5. Permutation tests and collinearity diagnostics for the Hellinger-based redundancy analysis (RDA).

Panel A. Overall RDA model

Overall model	R^2	Adjusted R^2	Permutation P	Interpretation
Selected 15 genera ~ six water-quality variables	0.7065	0.4864	0.016	Significant overall association

Panel B. Axis-wise permutation tests

Axis	Df	Variance	F	Raw P	Explained total variation (%)	Interpretation
RDA1	1	0.027290	16.314	0.014	59.85	Significant
RDA2	1	0.003060	2.058	0.761	6.71	Not significant
RDA3	1	0.001102	0.823	0.961	—	Not significant
RDA4	1	0.000392	0.322	0.999	—	Not significant
RDA5	1	0.000265	0.237	0.999	—	Not significant
RDA6	1	0.000104	0.101	0.999	—	Not significant

Panel C. Marginal tests for individual environmental variables, BH-adjusted q values and VIF

Variable	Df	Variance	F	Raw P	BH q	VIF	FDR result	Collinearity diagnostic
SS	1	0.000915	0.547	0.577	0.577	1.933	Not significant	Acceptable VIF (<5)
NH_4^+-N	1	0.002964	1.772	0.165	0.486	11.960	Not significant	VIF > 10
NO_3^--N	1	0.001677	1.003	0.362	0.543	5.853	Not significant	Elevated VIF (5–10)
NO_2^--N	1	0.002591	1.549	0.241	0.486	2.251	Not significant	Acceptable VIF (<5)
PO_4^{3-}-P	1	0.000987	0.590	0.560	0.577	2.311	Not significant	Acceptable VIF (<5)
Chl-a	1	0.002481	1.483	0.243	0.486	7.589	Not significant	Elevated VIF (5–10)

Note. Genus-level relative abundances were Hellinger transformed and the six environmental variables were z-score standardized. Permutation tests used 999 permutations. Benjamini–Hochberg adjustment was applied only to the six marginal environmental-variable tests; axis-wise P values were not included in that adjustment. The overall model was significant (P = 0.016), and only RDA1 was significant (P = 0.014). None of the six variables showed a significant independent marginal effect after correction. VIFs indicated strong collinearity for NH_4^+-N (11.960) and elevated collinearity for NO_3^--N (5.853) and Chl-a (7.589).

Table S6. Summary of the statistical analysis of PICRUSt2-predicted KEGG Level 3 pathways.

Analysis item	Result
Total Level 3 pathways provided	328
All-zero or invariant pathways excluded	22
Pathways tested using the Kruskal-Wallis test	306
Pathways with raw $P < 0.05$	59
Pathways significant after BH correction ($q < 0.05$)	0
Minimum raw P	0.013599
Minimum BH-adjusted q	0.190888
Environmentally relevant pathways displayed in Figure S5	16

Note. Predicted pathway abundances were converted to within-sample relative abundances before treatment means were calculated. Of 328 Level 3 pathways, 22 all-zero or invariant pathways were excluded, leaving 306 pathways for Kruskal-Wallis testing (df = 4). Raw P values were adjusted jointly using the Benjamini-Hochberg procedure. No pathway remained significant at $q < 0.05$. Complete pathway-level treatment means, Kruskal-Wallis H statistics, raw P values and BH-adjusted q values are provided in Supplementary Data S1.
