# Supplementary material for: Effects of Bellamya Stocking Density on Water Quality and Bacterial Community Responses in Aquaculture Effluent
Source: Microorganisms. 2026 Aug 17;14(8):1813. doi: 10.3390/microorganisms14081813 (PMC13515823; doi:10.3390/microorganisms14081813)
Supplement: Supplementary file 1 [file microorganisms-14-01813-s001.zip › Supplementary drawing of materials.pdf]

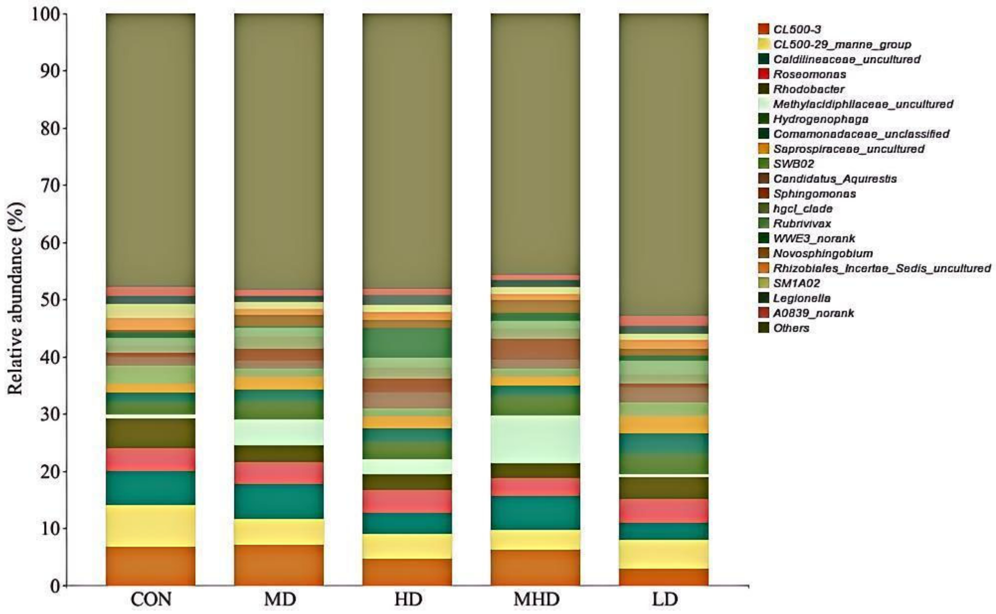

Figure S1. Genus-level bacterial community composition among Bellamya stocking-density treatments. Bars show treatment-level mean relative abundances of the 20 most abundant genus-level taxa in the normalized OTU table; all remaining taxa were grouped as “Others”.

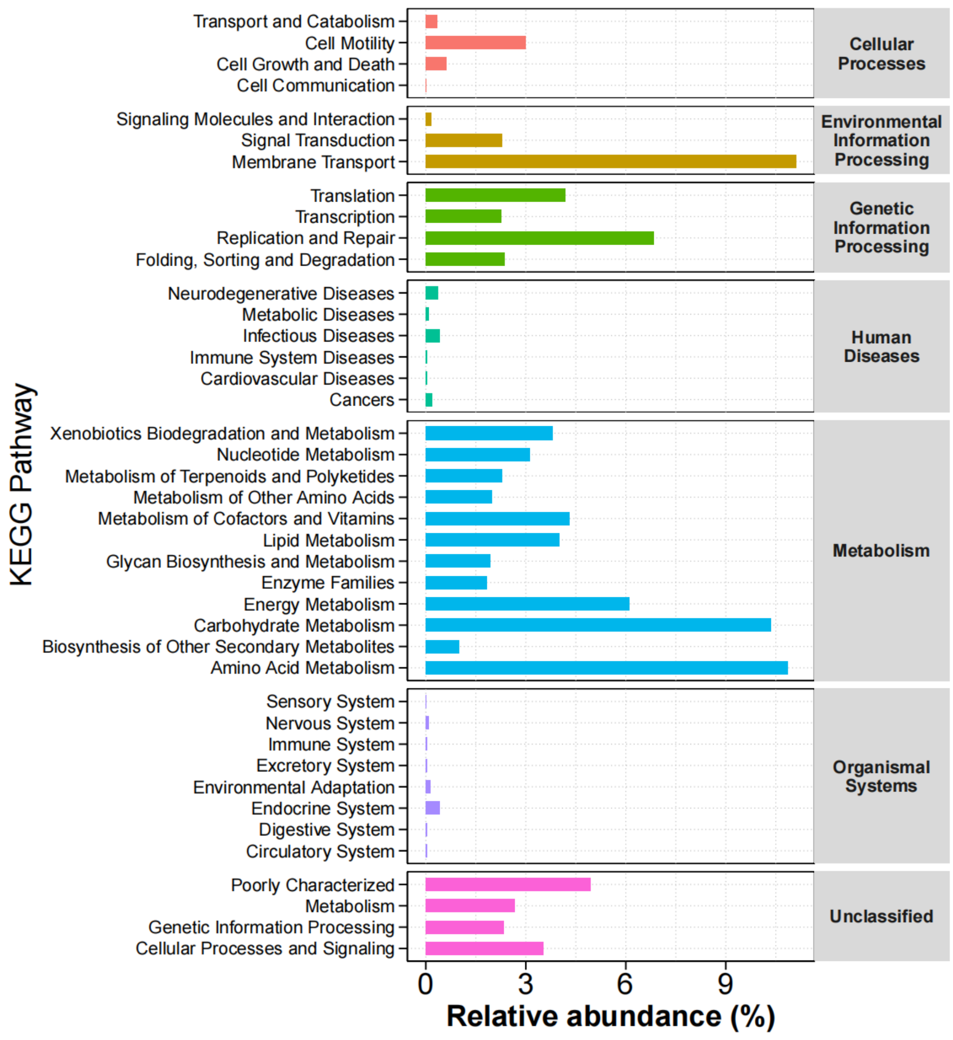

Figure S2. PICRUSt2-predicted KEGG Level 2 functional composition. Bars show the mean within-sample relative abundance of predicted functional categories across the 15 samples. These values are 16S-based predictions and do not represent direct measurements of genes, transcription, enzyme activity or pathway flux.

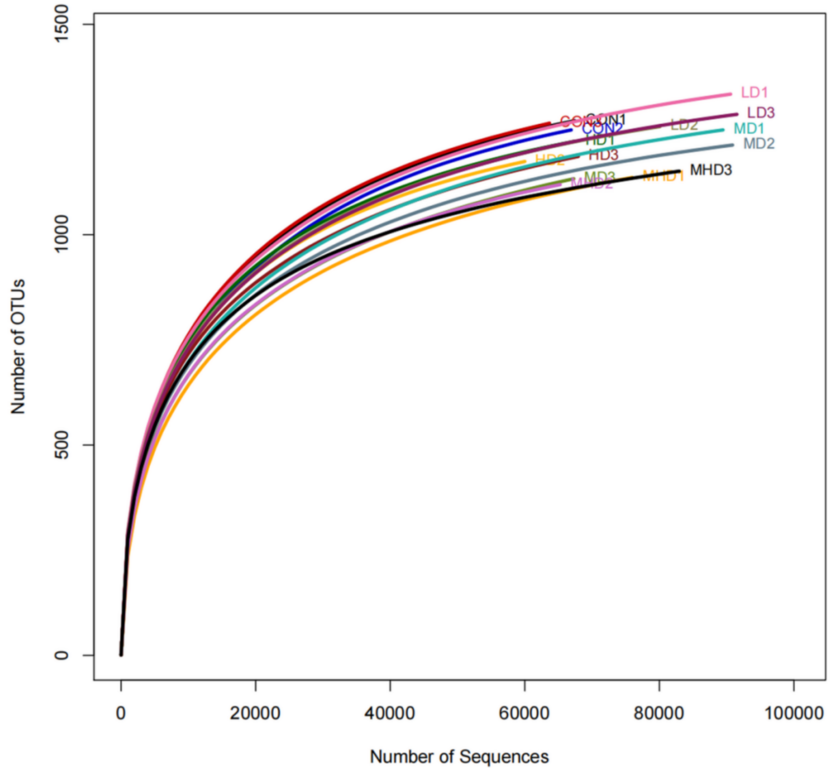

Figure S3. Rarefaction curves for bacterial OTUs clustered at 97% sequence similarity. Each curve represents one sample; the x-axis indicates sequencing depth and the y-axis observed OTUs. Curves approaching a plateau indicate adequate sequencing coverage.

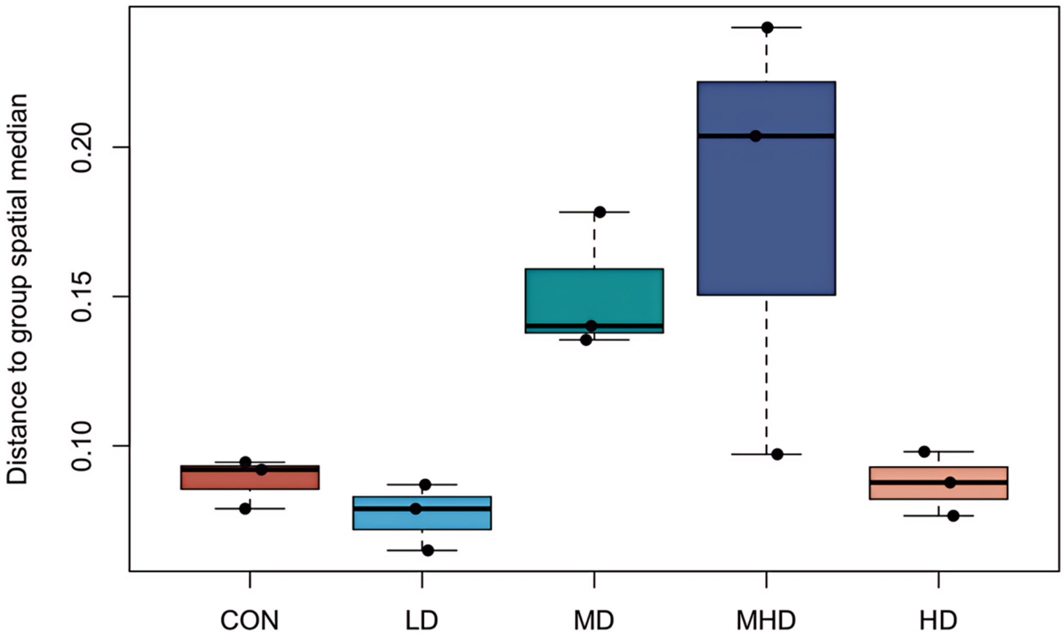

Figure S4. Multivariate dispersion of bacterial communities based on Bray–Curtis dissimilarity. Boxplots show sample distances to the treatment-group spatial median (*n* = 3 per treatment), calculated using betadisper (type = "median"); larger values indicate greater within-group dispersion. PERMDISP: $F_{4,10}$ = 4.99, P = 0.001 (999 permutations).

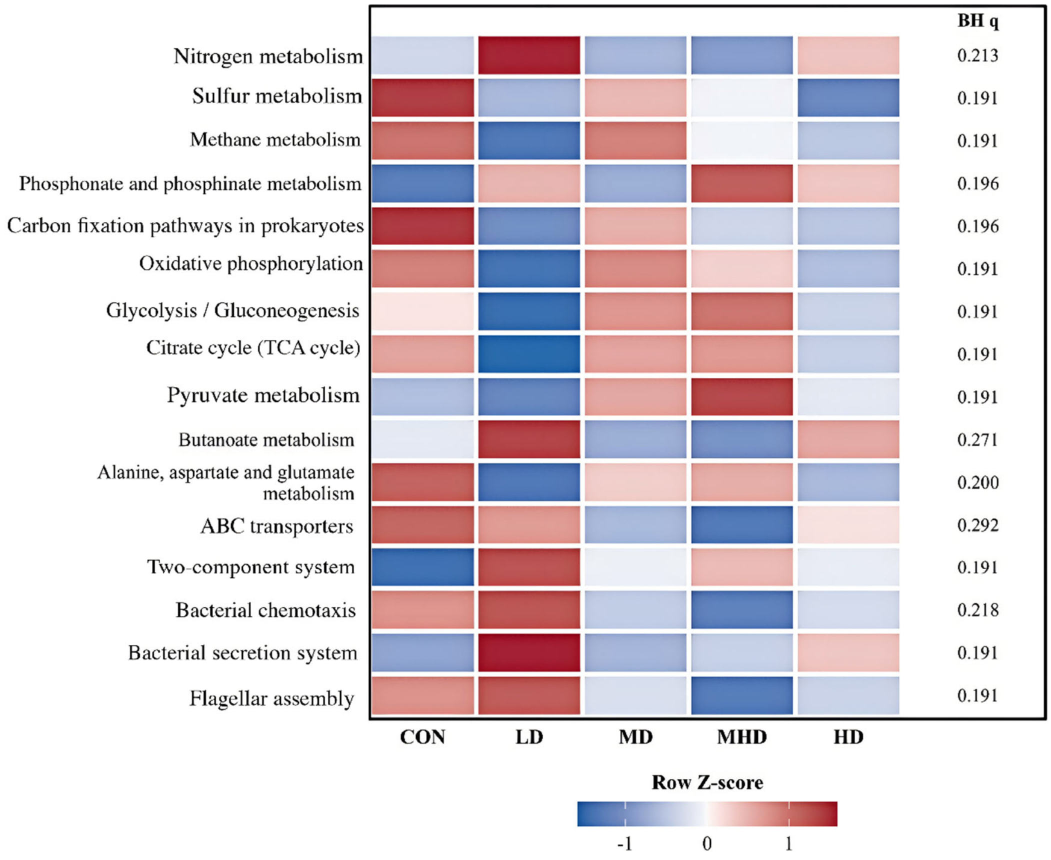

Figure S5. PICRUSt2-predicted KEGG Level 3 functional trends among *Bellamya* stocking-density treatments. Pathway abundances were converted to within-sample relative abundances, treatment means were calculated, and rows were standardized as Z-scores. The right-hand values are Benjamini–Hochberg-adjusted q values calculated across all 306 testable pathways. No pathway remained significant after FDR correction (all $q \geq 0.05$); the heatmap is therefore descriptive and does not demonstrate pathway activation or inhibition.
